# Milk Exosome-Based Delivery System for Probiotic Encapsulation That Enhances the Gastrointestinal Resistance and Adhesion of Probiotics

**DOI:** 10.3390/nu17050923

**Published:** 2025-03-06

**Authors:** Linlin Hao, Yinxue Liu, Ignatius Man-Yau Szeto, Haining Hao, Tai Zhang, Tongjie Liu, Huaxi Yi

**Affiliations:** 1 College of Food Science and Engineering, Ocean University of China, Qingdao 266003, China; haoqh5la@163.com (L.H.); liuyinxue_1121@126.com (Y.L.); haohaining1994@163.com (H.H.); tyzhang@stu.ouc.edu.cn (T.Z.); 2 Inner Mongolia Yili Industrial Group Co., Ltd., National Center of Technology Innovation for Dairy, Hohhot 010000, China; szeto@yili.com

**Keywords:** milk exosomes, probiotics, encapsulation, resistance, cell adhesion

## Abstract

The oral administration of probiotics is a promising strategy to regulate the host–intestinal flora balance and improve health. Nevertheless, adverse gastrointestinal (GI) conditions affect the activity of free native probiotics. In this study, a novel probiotic encapsulation system based on milk exosomes (mExos) and DSPE-PEG-PBA was developed. mExos acted as a shield to protect probiotics from harsh GI environments, and DSPE-PEG-PBA served as a bridge between mExos and probiotics. The coated probiotics were characterized by transmission electron microscopy (TEM), Fourier transform infrared spectroscopy (FT-IR), and intrinsic fluorescence spectra. The results showed three probiotics (*Akkermansia muciniphila* (AKK), *Bifidobacterium animalis* subsp. *lactis* BB-12 (BB12), and *Lactiplantibacillus plantarum* Q7 (Q7)) were coated with mExos@DSPE-PEG-PBA, with encapsulation rates of 90.37 ± 0.45%, 84.47 ± 1.22%, and 70.93 ± 2.39%, respectively. This encapsulation not only preserved the growth activity of the probiotics but also provided robust protection against the detrimental effects of acidic pH, bile salts, and digestive enzymes. The encapsulated strains Q7, BB12, and AKK demonstrated survival rates of 80.99 ± 0.41%, 85.28 ± 0.20%, and 94.53 ± 0.26%, respectively, in an in vitro simulated GI environment. The mExos@DSPE-PEG-PBA-encapsulated probiotics exhibited enhanced hydrophobicity and auto-aggregation capacity, accompanied by a significant improvement in mucoadhesive properties, which collectively potentiated their colonization potential within the gastrointestinal tract. These findings substantiate the potential of mExos as an encapsulation platform for probiotics, providing valuable insights into the selection of exosomes as encapsulating agents to enhance probiotic viability and mucoadhesive capacity.

## 1. Introduction

The intestinal microbiota plays a crucial role in maintaining intestinal environment stability and host health [1,2]. An imbalance in gut microbiota may lead to various diseases, such as inflammatory bowel disease (IBD), obesity, and even cancer [3,4]. Probiotics are living microorganisms beneficial to host health. The introduction of probiotics into the intestine can inhibit the colonization of pathogenic bacteria, regulate the intestinal microbiota balance, and enhance immune function [3,4]. For instance, *Bifidobacterium animalis* subsp. *lactis* BB-12 (BB12) can inhibit the adhesion of pathogenic bacteria in the intestine, reduce the apoptosis of intestinal epithelial cells, and improve dextran sodium sulfate (DSS)-induced colitis [5]. *Lactobacillus plantarum* Q7 (*L. plantarum* Q7) was reported as a probiotic to repair liver and kidney damage by remodeling intestinal flora and regulating the intestinal barrier [6]. *Akkermansia muciniphila* (AKK) improved metabolic fatty liver disease associated with a high-fat diet by regulating the gut microbiota and intestinal barrier by increasing the quantity of anti-inflammatory cytokines and Muc5ac [7,8]. However, harsh gastrointestinal (GI) environments have adverse effects on the growth and activity of probiotics [9,10,11]. Therefore, maintaining high levels of activity and survival rates among probiotics is vital. To solve these problems, microencapsulation has become one of the most common methods for increasing the probiotic survival rate under complex gastrointestinal conditions [12]. Probiotics are packed into food-grade sealed microcapsules. These capsules degrade and release probiotics when exposed to specific conditions [13]. This method can improve probiotic survival to a certain extent, but problems still exist, including complex operations, the difficulty in controlling particle size, the leakage of probiotics, and low efficiency in vivo [13,14]. As such, using a simple probiotic encapsulation system to effectively protect probiotics from adverse GI environments is necessary.

The utilization of nanoparticles for coating probiotics is an emerging technology [15]. Wei et al. developed mesoporous silica nanoparticles (MSNPs) [16]. Probiotics encapsulated by MSNPs exhibit resistance against simulated gastric juices and antibiotics in vitro, suggesting that MSNPs could potentially shield probiotics from gastric juices. However, the use of inorganic nanoparticles in food is limited due to their intrinsic toxicity and low solubility. Exosomes (Exos) are nanoscale vesicles released from host cells and hold promise as effective nanodelivery carriers [17]. Milk exosomes (mExos) can transport proteins and nucleic acids to the intestine and protect them from degradation and digestion. mExos can regulate the intestinal microflora in mice, promote intestinal mucus production, and repair disease-induced intestinal barrier damage [18,19]. Moreover, mExos play a crucial physiological role in maintaining normal intestinal function and are expected to become a new generation of prebiotics [20,21,22]. The hydrophobic lipid bilayer of mExos allows them to protect the embedded cargo. Zhang et al. discovered that an mExo-based drug encapsulation and delivery system exhibited controllable drug release rates, biocompatibility, and therapeutic efficacy [23]. In order to achieve superior encapsulation and delivery efficacy, specific ligands were typically modified on the Exo membrane surface. Kim et al. successfully modified the exosome membrane by combining aminoethylanisamide with distearoyl phosphatidyl ethanolamine-polyethylene glycol (DSPE-PEG) [24]. Therefore, exosomes modified with DSPE-PEG-based ligands hold great potential for delivery [25]. However, the modification of exosomes is primarily utilized for enhancing drug bioavailability. There are few reports on exosomes for the enhancement of probiotic tolerance in the GI tract. It was found that phenylboronic acid (PBA) could be bonded with cis-1,2/1,3-diols to form borate esters [26]. The surface of bacteria cells contains various polyhydroxy substances, such as glucose, sialic acid, and glycoprotein, which could be combined with PBA. These substances have made PBA-based polymers promising materials in the field of biomedical engineering [27,28,29]. The PBA-modified silver nanoparticles designed by Wang et al. exhibited exceptional bacterial binding capability [30]. Therefore, the surface modification of mExos using distearoyl phosphatidyl ethanolamine-polyethylene glycol-phenylboric acid (DSPE-PEG-PBA) might facilitate the recognition and binding of exosomes to bacteria, thereby enabling the encapsulation of probiotics by exosomes. Significantly, mExos have a natural affinity with and post-modification tendency toward probiotics, which provides more possibilities for the protection and delivery of probiotics.

Here, we developed a simple and effective encapsulation system (mExos@DSPE-PEG-PBA@Probiotics) to enhance probiotic resistance to GI environments. In this study, we first constructed a modified milk exosome system (mExo@DSPE-PEG-PBA), followed by the development of a linkage between mExo@DSPE-PEG-PBA and probiotics through an o-diol click reaction to form a protective “shield”. Experimental results demonstrated that the preparation method for mExo-coated probiotics could be applied to various strains, such as AKK, BB12, and *L. plantarum* Q7. Notably, with the protection of exosomes, mExo-coated probiotics could resist harsh GI environments and effectively adhere to intestinal cells. In brief, our findings indicate that mExo-coated probiotics exhibit superior protective effects for probiotic preservation. This design strategy provides a promising approach for developing functional exosomes and exosome-based platforms.

## 2. Materials and Methods

### 2.1. Isolation and Identification of Exosomes

Bovine raw milk was purchased from the Qingdao Animal Husbandry Research Institute, with the permission of the farm manager (Qingdao, China). Milk exosomes were obtained according to the report of Tong et al. [31]. The protein concentration of the milk exosomes was determined with a BCA kit (Beyotime Biotechnology Co., Ltd., Shanghai, China). The particle size distribution of the mExos was determined by Zetasizer Nano ZS (Malvern Instruments Ltd., Malvern, UK), according to the method of Cui et al. [17]. The particle morphology of the mExos was determined by transmission electron microscopy (TEM) (Hitachi, Ltd., Tokyo, Japan).

The mExo surface proteins were determined by Western blotting (WB) [17,32]. Samples were subjected to SDS-PAGE and then transferred to a polyvinylidene fluoride (PVDF) membrane. The primary antibodies were incubated with the samples overnight at 4 °C. The specific proteins were incubated with an enzyme-labeled secondary antibody and detected by chemiluminescence using an ECL Prime kit (Sigma-Aldrich, St. Louis, MO, USA). A fully automatic chemiluminescence fluorescence imager (Tanon, Shanghai, China) was used to characterize the proteins. Anti-TSG101 (ab225877) and anti-calnexin (ab133615) were purchased from Abcam (Cambridge, UK). Anti-CD81 (GTX101766) and anti-CD9 (GTX76185) were purchased from Gene Tex (Irvine, CA, USA).

### 2.2. Surface Modification of the mExos

To obtain PBA-modified exosomes, the mExos were mixed with DSPE-PEG-PBA (Xian Qiyue Trading Co., LTD., Xian, China), and the mixture was incubated under magnetic stirring at 4 °C for 48 h without light. The final concentrations of both the exosomes and DSPE-PEG-PBA were 1 mg/mL. The mExo@DSPE-PEG-PBA was then centrifuged (Hitachi, Ltd., Tokyo, Japan) at 4 °C and 100,000× *g* for 1 h to remove the free DSPE-PEG-PBA. The precipitates were redissolved with PBS and preserved at 4 °C [33].

### 2.3. Construction of mExo@DSPE-PEG-PBA-Coated Probiotics

Three probiotic strains, AKK, *L. plantarum* Q7, and BB12, were used in this study. *L. plantarum* Q7 was previously isolated from yak milk, and the BB12 strain was purchased from ATCC [7]. The AKK strain was provided by China Agricultural University (Beijing, China). *L. plantarum* Q7 and BB12 were cultured in de Man, Rogosa, and Sharpe (MRS) at 37 °C for 48 h before use. AKK was cultured in a brain heart infusion broth (BHI) medium (Hopebio, Qingdao, China), supplemented with 0.05% L-Cysteine hydrochloride anhydrous (Macklin, Shanghai, China) under anaerobic conditions. The fermentation broth was centrifuged at 8000 rpm for 5 min at 4 °C. The precipitation was collected, washed twice with sterile PBS, and resuspended. The probiotics (1 × 10^8^ CFU mL^−1^) were incubated with mExo@DSPE-PEG-PBA (500 µg mL^−1^) under oscillating conditions for 30 min. The mExo@DSPE-PEG-PBA was assembled on probiotics through a boronic acid vicinal-diol-based click reaction [34].

### 2.4. Characterization of mExo@DSPE-PEG-PBA@Probiotics

The particle sizes of mExo@DSPE-PEG-PBA and mExo@DSPE-PEG-PBA@Probiotics were determined by Zetasizer Nano ZS (Malvern ZS 90, UK) according to the method of Cui et al., with minor modifications [17]. The surface zeta potential of the mExos, DPEG-PEG-PBA, and mExo@DSPE-PEG-PBA were measured by Zetasizer Nano ZS (Malvern ZS 90, UK). The morphology of mExo@DSPE-PEG-PBA and mExo@DSPE-PEG-PBA@Probiotics was determined by TEM. The surface proteins of mExo@DSPE-PEG-PBA were determined by Western blotting.

The mExos, mExo@DSPE-PEG-PBA, and mExo@DSPE-PEG-PBA@probiotics were analyzed using Fourier transform infrared spectroscopy (FTIR) (Thermo Fisher Scientifi Ltd., Waltham, MA, USA). The probiotics before and after mExo@DSPE-PEG-PBA coating were analyzed using fluorescence spectra [35]. The fluorescence intensity of PBA moiety (E_x_ = 302 nm, E_m_ = 388 nm) was measured. Both the excitation and emission bandwidths were set at 5 nm.

### 2.5. Determination of Probiotic Embedding Rate

The embedding rate of probiotics was determined by flow cytometry (Becton, Dickinson and Company, Franklin Lakes, NJ, USA) according to the method of Geng et al. [36]. The mExos were fluorescently labeled with Dio. An equal volume of Dio solution was added to the mExo solution, followed by incubation at 25 °C in the dark for 1 h. Excess dye was removed by ultracentrifugation at 4 °C and 100,000× *g*, and the labeled mExos were washed twice with PBS. The fluorescently labeled mExos were then used to encapsulate the probiotics following the procedures outlined in Section 2.2 and Section 2.3. The DiO-labeled exosomal-coated bacteria were determined by flow cytometry (BD FACSVerse, Brea, CA, USA). Native bacteria were used as a control.

### 2.6. Influence of mExos@DSPE-PEG-PBA on Probiotic Growth

The cultured bacteria were collected by centrifugation (6000 rpm, 5 min) and rinsed with PBS, as described in Section 2.3. The native bacteria were diluted with PBS to the same concentration as the coated bacteria. Serial dilutions of the probiotic suspensions were made in PBS and plated on agar plates. *L. plantarum* Q7 and mExo@DSPE-PEG-PBA@Q7 were cultured at 37 °C for 48 h. BB12, AKK, mExo@DSPE-PEG-PBA@BB12, and mExo@DSPE-PEG-PBA@AKK were cultured at 37 °C in an anaerobic chamber for 48 h. The number of viable cells was expressed as CFU/mL [37].

### 2.7. Evaluation of the GI Tolerance of mExo@DSPE-PEG-PBA@probiotics

The acid and bile tolerance were investigated according to Lu et al., with minor modifications [38]. mExo@DSPE-PEG-PBA@Q7, mExo@DSPE-PEG-PBA@BB12, mExo@DSPE-PEG-PBA@AKK, *L. plantarum* Q7, BB12, and AKK were suspended in a culture medium at pH 3.0 or 0.3% bile salt and incubated at 37 °C for 3 h. The survival rate formula was as follows:

(1) $$\mathrm{Survival}\;\mathrm{rate}\;(\%)=\frac{\log\;\mathrm{CFU}\;N_{t}}{\log\;\mathrm{CFU}\;N_{0}}\times 100\%$$

where N_0_ and N_t_ are the total viable counts before and after treatment, respectively.

Simulated gastroenteric fluid (SGF) and simulated intestinal fluid (SIF) were prepared according to the previous method, with minor modifications [34]. SGF (9 g/L NaCl, 3.2 g/L pepsin), which adjusted the pH to 2 with 1 M HCl, and SIF (6.8 g/L KH_2_PO_4_, 10 g/L trypsin, 0.3 mg/mL bile salt), which adjusted the pH to 6.8, were prepared and filtered through a 0.22 μm filter before the experiment. Next, 0.1 mL of bacteria were immersed in 1 mL of SGF and continuously shaken at 140 rpm/min for 0.5 h at 37 °C. Then, 0.5 mL of the sample of the digested SGF was transferred to 0.5 mL of SIF and continuously shaken at 140 rpm/min for 2 h at 37 °C. The content of bacteria was measured by the standard plate counting method. The survival rate of the bacteria was calculated using Equation (1).

### 2.8. Determination of mExo@DSPE-PEG-PBA@probiotic Hydrophobicity and Self-Polymerization

Surface hydrophobicity was determined according to the previous method [38]. First, 3 mL of the sample solution was mixed with 1 mL of chloroform, and the two-phase mixed system was swirled and shaken for 2 min. The absorbance value of the water phase in the mixed solution was measured at OD600. The hydrophobic rate was calculated as follows:

(2) $$\mathrm{Hydrophobicity}=[1-(\frac{A_{t}}{A_{0}})]\times 100\%$$

where A_t_ is the absorption value of the water phase after 30 min and A_0_ is the initial value.

Self-aggregation was assayed according to the method of Bu et al. [39]. The absorption value A_1_ was measured at 600 nm. Then, 4 mL of the sample solution was vortexed for 1 min and left at room temperature for 3 h. The absorption of the supernatant was measured at 600 nm. The self-aggregation rate was calculated by the following formula:

(3) $$\mathrm{Self}\text{-}\mathrm{aggregation}=[1-(\frac{A_{2}}{A_{1}})]\times 100\%$$

where A_1_ and A_2_ are the absorbance values at 0 min and 3 h, respectively.

### 2.9. Effect of mExo@DSPE-PEG-PBA@probiotics on Cell Viability

Caco-2 cells were cultured in a high-glucose DMEM medium supplemented with 10% fetal bovine serum, 100 U/mL of penicillin, and 100 μg/mL of streptomycin. When the density of the Caco-2 cells reached 1 × 10^4^ cells/well, mExo@DSPE-PEG-PBA@probiotics were added to the cell culture medium and incubated for 24 h. The Caco-2 cells were collected by ultracentrifugation and washed with PBS three times. The CCK-8 (GlpBio, Shanghai, China) reagent was added and incubated at 37 °C for 3 h. The absorbance value was determined at 450 nm. Cell viability was calculated by the following formula:

(4) $$\mathrm{Cell}\;\mathrm{viability}=\frac{A_{s}-A_{b}}{A_{c}-A_{b}}\times 100\%$$

where A_s_ is the absorbance of the experimental group, A_b_ is the absorbance of the blank, and A_c_ is the absorbance of the control.

### 2.10. Cell Adhesion Assay

The adhesion of the probiotics to Caco-2 cells was performed as previously reported, with some modifications [40,41]. The probiotic concentration of each sample was diluted to 1× 10^7^ CFU/mL with DMEM medium and incubated with Caco-2 cells at 37 °C for 4 h. After 4 h, the DMEM medium in the pore plates was discarded, and the pores were washed twice with PBS to remove the adhesion bacteria. The monolayer of the cells was digested with trypsin solution, and the cells were counted by the blood cell counting plate method. The number of probiotic cells attached to the Caco-2 cells was counted by inoculation in MRS or BHI agar. The adhesion of the probiotics was expressed as the number of viable bacteria adhering to 100 Caco-2 cells. Each experiment was conducted in triplicate.

### 2.11. Statistical Analysis

Each experiment was performed at least three times. All the experimental data were presented as means ± standard deviation. An independent-samples *t*-test was used to compare the statistical differences between two groups, and one-way ANOVA and the *t*-test were performed by GraphPad Prism 8.0.2 software. *p* < 0.05 was considered to be significantly different.

## 3. Results

### 3.1. Isolation and Identification of mExos

**Figure 1:**
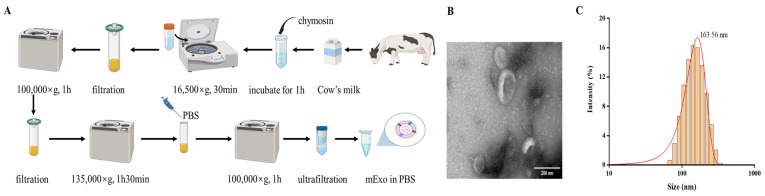
Preparation and characterization of mExos. (**A**) Schematic of the extraction of mExos from milk. (**B**) TEM image of mExos. (**C**) Size distribution of mExos. Scale bar, 200 nm.

Milk is a complex and heterogeneous matrix; thus, it poses challenges in the separation and purification of mExos. Based on our previous study [31], we employed a chymosin treatment combined with ultracentrifugation and ultrafiltration to achieve mExo isolation (Figure 1A). The TEM analysis revealed that the mExos had a cup-shaped spherical structure, which was consistent with previous reports (Figure 1B) [31]. TME can directly observe the morphology of exosomes, thus providing evidence of exosome existence. Furthermore, nanoparticle size analysis demonstrated that the size distribution of the mExos ranged from 50 nm to 300 nm, with an average particle size of 163.56 ± 1.57 nm (Figure 1C), which aligned well with our previous findings [31].

### 3.2. Preparation and Characterization of mExo@DSPE-PEG-PBA

**Figure 2:**
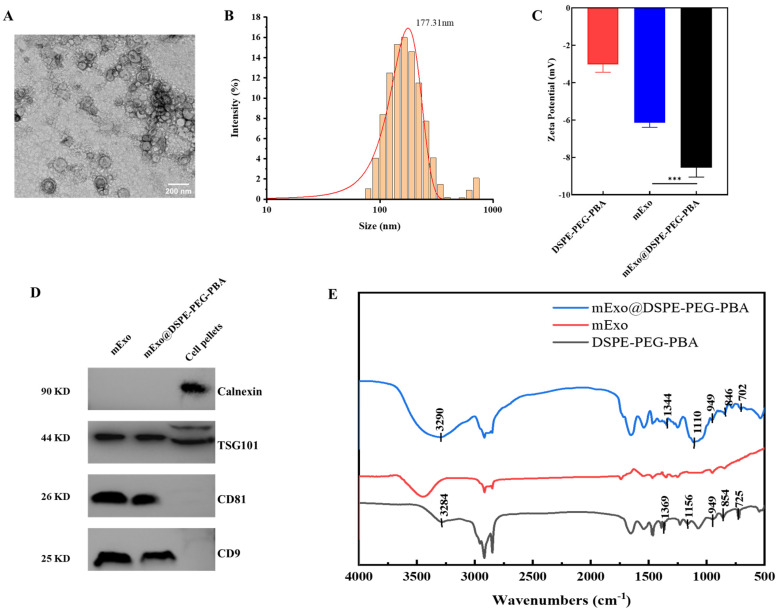
Characterization of mExo@DSPE-PEG-PBA. (**A**) TME images of mExo@DSPE-PEG-PBA. Scale bar, 200 nm. (**B**) Size distribution of mExo@DSPE-PEG-PBA. (**C**) The zeta potential of DSPE-PEG-PBA, mExos, and mExo@DSPE-PEG-PBA. (**D**) mExo markers and calnexin analyzed with Western blotting. (**E**) FITR analysis of mExo@DSPE-PEG-PBA. *** *p* < 0.001.

TEM was used to verify the morphology and size of mExo@DSPE-PEG-PBA and to analyze the modification of the mExos by DSPE-PEG-PBA by particle size and zeta potential. The surface morphology of mExo@DSPE-PEG-PBA displayed typical exosomal characteristics, and the average particle size was increased to 177.31 ± 1.99 nm, which indicated that the surface of the mExos was modified by PBA successfully (Figure 2A,B). In order to better describe the modification of the mExos by DSPE-PEG-PBA, we measured the changes of the mExo zeta potential before and after modification. As shown in Figure 2C, the zeta potential of DSPE-PEG-PBA, the mExos, and mExo@DSPE-PEG-PBA were −3.02 ± 0.42 mV, −6.14 ± 0.25 mV, and −8.56 ± 0.50 mV, respectively. mExo@DSPE-PEG-PBA showed a more negative potential, suggesting that PBA had effectively decorated onto the mExos. Furthermore, WB analysis was used to detect proteins that were specifically expressed or not present in the exosomes. Cell pellets from human embryonic kidney 293T cells and HepG2 human hepatoma cells were used as the positive control. Calnexin is a non-exosome marker and was used as a negative control in our study in the identification of mExos. In addition, the mExos contained abundant exo-related proteins, such as tetraspanins (CD9, CD81), ESCRT-I/II/III(TSG101), etc. The WB analysis results showed calnexin was not found in the mExos (Figure 2D). The separation and purification process did not affect the integrity of the mExo membrane proteins [42]. Notably, DSPE-PEG-PBA modification did not affect the specific protein level of the mExos.

The conjunction characterization of mExo@DSPE-PEG-PBA was investigated using FITR spectra. As shown in Figure 2E, DSPE-PEG-PBA exhibited the stretching vibration of hydrogen-bonded -OH at 3284 cm^−1^. In addition, B-O and B-C characteristic peaks were observed at 1369 cm^−1^ and 1156 cm^−1^, respectively. The characteristic peaks of the benzene ring appeared at 725, 854, and 949 cm^−1^. In the infrared spectrum of mExo@DSPE-PEG-PBA, the characteristic peaks of hydrogen bonds, –OH, B-O, and B-C, appeared at 3290 cm^−1^, 1344 cm^−1^, and 1110 cm^−1^, respectively. The benzene ring fingerprint peak was found at 650–950 cm^−1^. These results indicated that DSPE-PEG-PBA was attached to the phospholipid bilayer of the mExos via hydrophobic DSPE.

### 3.3. Design and Characterization of mExo@DSPE-PEG-PBA@Probiotics

**Figure 3:**
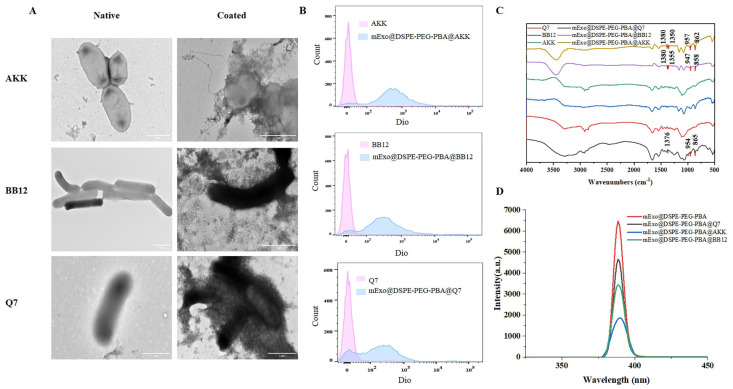
Characterization of mExo@DSPE-PEG-PBA@Probiotics. (**A**) TEM images of native probiotics and coated probiotics. Scale bar, 1 µm. (**B**) Flow cytometry histograms of Dio-labeled mExo@DSPE-PEG-PBA@Probiotics. Native probiotics were used as the control. (**C**) FTIR spectra of native and coated probiotics. (**D**) Fluorescence spectra of mExo@DSPE-PEG-PBA and mExo-embedded probiotics.

PBA might covalently bind to the polysaccharide of probiotics through the click reaction. Thus, mExos were deposited on the surface of the probiotics, forming a protective barrier. In Figure 3A, it can be clearly seen that the mExos are connected to *L. plantarum* Q7, BB12, and AKK. The binding of the mExos to the surface of the probiotics was further confirmed. The coating rate of the mExo-coated probiotics was analyzed by flow cytometry. The coating rates of mExo@DSPE-PEG-PBA@AKK, mExo@DSPE-PEG-PBA@BB12, and mExo@DSPE-PEG-PBA@Q7 were 90.37 ± 0.45%, 84.47 ± 1.22%, and 70.93 ± 2.39%, respectively (Figure 3B). This showed that mExo@DSPE-PEG-PBA had an excellent encapsulation effect on these three strains, but the encapsulation rate was slightly different.

Characterization of the mExo@DSPE-PEG-PBA@probiotics conjunctions was further conducted using FITR spectra and fluorescence spectra to elucidate its synthesis process. The structure of mExo@DSPE-PEG-PBA@Probiotics was characterized by FITR and fluorescence spectroscopy. As shown in Figure 3C, the B-O characteristic peak appeared at 1355–1310 cm^−1^, which suggests that the mExos modified by PBA were bound to the bacterial surface. The appearance of benzene ring fingerprint peaks at 650–950 cm^−1^ further confirm this result [43]. Since the interaction between PBA and diol could induce the fluorescence quenching of PBA, the fluorescence changes of PBA before and after the embedding probiotics were measured by a fluorescence spectrophotometer [35]. The more PBA bound to the diol, the more the fluorescence intensity of PBA decreased. As shown in Figure 3D, when mExo@DSPE-PEG-PBA was combined with *L. plantarum* Q7, BB12, and AKK, the fluorescence intensity decreased. mExo@DSPE-PEG-PBA@AKK had the lowest fluorescence intensity, and mExo@DSPE-PEG-PBA@Q7 had the highest fluorescence intensity. An explanation for this could be that the more PBA binds to o-diol, the stronger the embedding effect.

### 3.4. Effects of mExo@DSPE-PEG-PBA on Probiotic Growth

**Figure 4:**
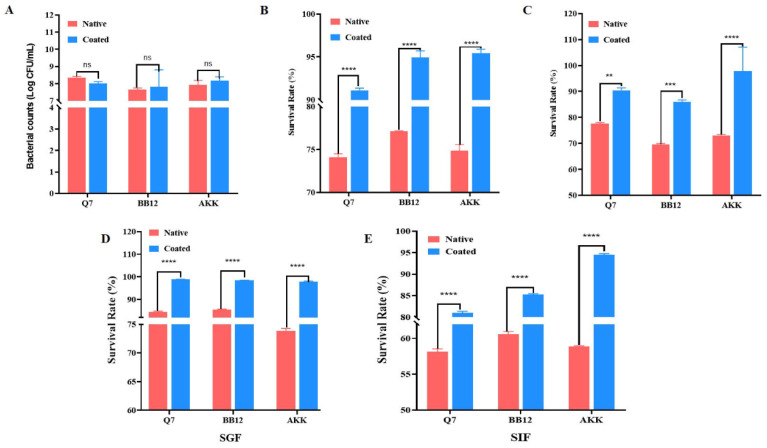
Effect of mExo@DSPE-PEG-PBA on the growth and survival of probiotics. (**A**) Growth of *L. plantarum* Q7, BB12, and AKK before and after embedding. (**B**) Acid resistance of mExo@DSPE-PEG-PBA@Probiotics. (**C**) Bile salt resistance of mExo@DSPE-PEG-PBA@Probiotics. (**D**) SGF survival rate of mExo@DSPE-PEG-PBA@Probiotics. (**E**) SIF survival rate of mExo@DSPE-PEG-PBA@Probiotics. ** *p* < 0.01, *** *p* < 0.001, **** *p* < 0.0001. ns is not significantly different.

As depicted in Figure 4A, there were no significant differences in the total number of *L. plantarum* Q7, BB12, and AKK strains before and after embedding. This indicates that mExo@DSPE-PEG-PBA did not affect the growth of the probiotics. In order to further explore the protective effect of mExo@DSPE-PEG-PBA on probiotics, we evaluated the survival of the probiotics under acidic and bile salt environments. The results showed that the survival rates of mExo@DSPE-PEG-PBA@Q7, mExo@DSPE-PEG-PBA@BB12, and mExo@DSPE-PEG-PBA@AKK were higher than those of the unembedded bacteria under acidic conditions. The embedded *L. plantarum* Q7, BB12, and AKK showed higher acid tolerance (Figure 4B). Under the condition of 0.3% bile salt, the survival rates of mExo@DSPE-PEG-PBA@Q7, mExo@DSPE-PEG-PBA@BB12, and mExo@DSPE-PEG-PBA@AKK were higher than those of unembedded probiotics (Figure 4C). These results indicate that the mExo@DSPE-PEG-PBA@Probiotics system enhances the bile salt tolerance of probiotics.

It is vital for probiotics to survive during their transit through the GI tract [44]. The survival rates of *L. plantarum* Q7, BB12, and AKK after gastrointestinal digestion before and after embedding were determined. In simulated gastric juices, the survival rates of mExo@DSPE-PEG-PBA-coated *L. plantarum* Q7, BB12, and AKK were significantly increased by 14.37%, 12.91%, and 23.95%, respectively (Figure 4D). The survival rates of the probiotics coated with mExo@DSPE-PEG-PBA were significantly improved during the digestion process. The final survival rates of mExo@DSPE-PEG-PBA@Q7, mExo@DSPE-PEG-PBA@BB12, and mExo@DSPE-PEG-PBA@AKK were 80.99 ± 0.41%, 85.28 ± 0.20%, and 94.53 ± 0.26%, respectively (Figure 4E). The mExo@DSPE-PEG-PBA@Probiotics system was able to resist the digestion of gastric and intestinal fluids.

### 3.5. Effect of mExo@DSPE-PEG-PBA@Probiotics on Cell Adhesion

**Figure 5:**
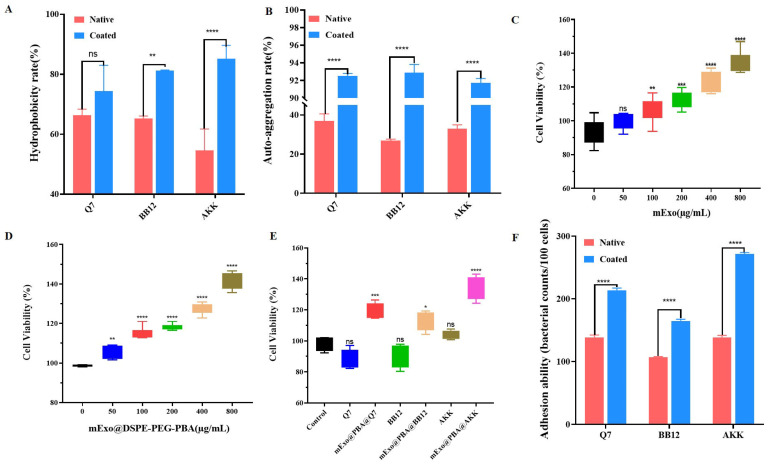
Adhesion of mExo@DSPE-PEG-PBA@Probiotics. Hydrophobicity (**A**) and self-aggregation (**B**) of three probiotics before and after embedding. Effect of mExos (**C**) and mExo@DSPE-PEG-PBA (**D**) on cell viability. Effects of mExo@DSPE-PEG-PBA@Probiotics on cell viability (**E**). Adhesion assays of mExo@DSPE-PEG-PBA@Probiotics (**F**). * *p* < 0.05, ** *p* < 0.01, *** *p* < 0.001, **** *p* < 0.0001. ns is not significantly different.

The hydrophobicity and self-aggregation ability of probiotics before and after embedding were measured to explore the potential of the intestinal colonization of probiotics. It was observed that the hydrophobicity of mExo@DSPE-PEG-PBA@Q7, mExo@DSPE-PEG-PBA@BB12, and mExo@DSPE-PEG-PBA@AKK was increased to 74.34 ± 8.62%, 81.26 ± 0.15%, and 85.21 ± 4.40%, respectively (Figure 5A). At the same time, the self-aggregation rates of mExo@DSPE-PEG-PBA@Q7, mExo@DSPE-PEG-PBA@BB12, and mExo@DSPE-PEG-PBA@AKK were 92.52 ± 0.27%, 92.90 ± 0.92%, and 91.73 ± 0.51%, respectively (Figure 5B). The increase in the self-aggregation rate of probiotics might be related to the embedding of the mExos, which increased the surface area of the probiotics.

The cytotoxicity of the mExos, mExo@DSPE-PEG-PBA, probiotics, and mExo@DSPE-PEG-PBA@probiotics were assessed by CCK-8 assay. Figure 5C shows that the mExos and mExo@DSPE-PEG-PBA were non-toxic to cells. With the increase in the mExo and mExo@DSPE-PEG-PBA concentration, the activity of the Caco-2 cells increased, which indicated that the mExos and mExo@DSPE-PEG-PBA had good biosafety. As seen in Figure 5D, mExo@DSPE-PEG-PBA@probiotics had no toxic effects on the cells. The adherence properties of the free probiotics and mExo@DSPE-PEG-PBA@Probiotics in relation to the Caco-2 cells were investigated. In Figure 5E, it can be observed that the adhesion ability of mExo@DSPE-PEG-PBA@Probiotics has been improved. Compared with the free probiotics, the adhesion of mExo@DSPE-PEG-PBA@AKK, mExo@DSPE-PEG-PBA@Q7, and mExo@DSPE-PEG-PBA@BB12 was increased by 133 bacterial counts/100 cells, 75 bacterial counts/100 cells, and 58 bacterial counts/100 cells, respectively.

## 4. Discussion

As a promising natural nanodelivery carrier, mExos exhibit excellent biocompatibility and gastrointestinal tolerance, demonstrating the potential to encapsulate and protect probiotics. In this study, the mExo surface was engineered with DSPE-PEG-PBA to target polysaccharides on the probiotic surface, thereby enabling exosome deposition on the probiotic surface and the formation of a protective exosomal shield. DSPE-PEG-PBA was utilized for the surface modification of the mExos. Lipophilic DSPE was inserted into the phospholipid bilayer of the exosomes by self-assembly. The flexible connecting arm of PEG-PBA was exposed on the surface of the mExos. The surface morphology of mExo@DSPE-PEG-PBA displayed typical exosomal characteristics, and the average particle size and the zeta potential suggested that PBA was effectively coated onto the mExos. Notably, DSPE-PEG-PBA modification did not affect the specific protein levels of the mExos. This is similar to a report in the existing literature, which reported that surface modification did not affect the expression of exosomal marker proteins [17,24]. The FITR results indicated that DSPE-PEG-PBA was attached to the phospholipid bilayer of the mExos via hydrophobic DSPE.

PBA might covalently bind to the polysaccharide of probiotics through the click reaction. Through the reaction of polysaccharides with boric acid, hydrophobic molecules containing boric acid could attach to biopolymers [45]. Thus, the mExos were deposited on the surface of the probiotics, forming a protective barrier. This showed that mExo@DSPE-PEG-PBA had an excellent encapsulation effect on these three strains, but the encapsulation rate was slightly different, which might be related to the types of polysaccharides on the surface of the probiotics. It was also proved that boric acid derivatives could effectively bind with polysaccharides on the surface of bacterial cell walls. This targeting approach based on PBA exhibited specificity and a high capture rate [46]. Ye et al. reported that boric acid-functionalized CdSe/CdS/ZnS quantum dots could specifically detect lipopolysaccharide (LPS) on the surface of Gram-negative bacteria [47]. Interestingly, certain boric acid-based nanoprobes exhibited an intriguing ability to select Gram-positive bacteria [48]. Ayame et al. demonstrated that PBA displayed certain selectivity toward different bacteria [49]. The fluorescence changes in PBA before and after embedding probiotics were measured by a fluorescence spectrophotometer. The explanation for these results might be that the more PBA binds to o-diol, the stronger the embedding effect. Kim et al. proved that the reaction of PBA with o-diol was concentration-dependent [35].

We further investigated the impact of mExo@DSPE-PEG-PBA on probiotic growth under different environmental conditions. The results indicated that the mExo@DSPE-PEG-PBA@Probiotics system enhanced the pH and the bile salt tolerance of probiotics. Moreover, the results indicated that the mExo@DSPE-PEG-PBA@Probiotics system could resist the digestion of gastric and intestinal fluids. It was reported that curcumin encapsulated by mExos was more stable compared to free curcumin in in vitro digestion, which indicated that mExos could protect coated cargo against the harsh action of digestive juices [50].

The intestinal adhesion ability of probiotics was correlated with their hydrophobicity and self-aggregation, which facilitated probiotic survival in the GI tract [51]. The increase in the self-aggregation rate of probiotics might be related to the embedding of mExos, which increased the surface area of probiotics. Rajab et al. suggested that bacteria with a larger surface area had higher levels of self-aggregation and coaggregation [52]. It was reported that the adherence and aggregation of probiotics were closely associated. Zawistowska-Rojek et al. pointed out that the ability of Lactobacillaceae to self-aggregate was related to the adhesion capacity [53]. These results indicated that the mExo@DSPE-PEG-PBA@probiotics system enhanced the self-aggregation ability of probiotics, thereby facilitating their adhesion ability in the GI tract.

## 5. Conclusions

Milk exosomes, as natural nanoparticles, exhibited significant potential for encapsulating and delivering probiotics. A milk exosome-based delivery system for probiotic encapsulation was explored. mExo@DSPE-PEG-PBA could effectively envelop the surface of *Akkermansia muciniphila*, *B. animalis* subsp. *lactis* BB-12, and *L. plantarum* Q7, protect these probiotics from the adverse GI environment, and improve their adhesion ability. These findings suggest that the mExo@DSPE-PEG-PBA@Probiotics system could be developed as a new carrier, not only for embedding and delivering probiotics but also for enhancing probiotic viability, intestinal adhesion, and colonization.

## Data Availability

The data are available in the paper or from the authors upon request.
